# In vivo and in situ programming of tumor immunity by combining oncolytics and PD-1 immune checkpoint blockade

**DOI:** 10.1186/s40164-017-0075-4

**Published:** 2017-05-24

**Authors:** Eric Bartee, Zihai Li

**Affiliations:** Hollings Cancer Center, Department of Microbiology and Immunology, Medical University of South Carolina, 173 Ashley Ave, Charleston, SC 29425 USA

**Keywords:** Oncolytics, Checkpoint blockade, PD1

## Abstract

Blockade of the programmed cell death protein 1 (PD1) pathway is clinically effective against human cancers. Although multiple types of malignancies have been shown to respond to PD1 agents, only a small percentage of patients typically benefit from this treatment. In addition, PD1 therapy often causes serious immune-related adverse events. A recent study demonstrated that local, intra-tumoral, administration of modified oncolytic myxoma virus which expresses a truncated version of the PD1 protein resulted in both increased efficacy and reduced toxicity in a clinically relevant melanoma model.

Blockade of the PD1 pathway has produced impressive clinical results in many late stage cancer patients and is poised to fundamentally rewrite our concepts about cancer therapy [1]. PD1 is typically expressed on the surface of activate T cells. Prolonged engagement of PD1 with its primary ligand, programmed death ligand 1 (PDL1) (also known as B7-H1), results in long-term T cell exhaustion and a loss of functional immunity. This pathway likely evolved to limit pathogenic autoimmune reactions against normal tissues; however, it is frequently co-opted in cancers which overexpress PDL1 as a method of preventing anti-tumor immune responses. The resulting immuno-suppression limits effective immune surveillance allowing for tumor escape [2]. Blockade of the PD1/PDL1 pathway, using the FDA approved blocking antibodies nivolumab, pembrolizumab, atezolizumab, durvalumab, or avelumab, can have profound clinical effects in patients with an ongoing anti-tumor immune response. Unfortunately, it is largely ineffective in patients whose tumors are immunologically naïve, and systemic administration of these blocking antibodies also eliminates the normal function of the PD1 pathway which results in autoimmune disease. Discovering methods to improve response rates to PD1 therapy while limiting toxicities is therefore of critical importance.

One proposed solution to these problems is to supply PD1-blocking reagents directly to the tumor microenvironment. This allows for higher localized concentrations of PD1 blockade while limiting the potential for peripheral toxicities. This could be accomplished through direct intratumoral injection of αPD1 antibodies; however, a more attractive method is to incorporate PD1-blocking reagents into existing cancer therapies, such as oncolytic viruses. Unfortunately, while several groups have attempted to incorporate αPD1 scFv’s into oncolytic genomes, these recombinant viruses have generally displayed reduced overall efficacy compared to the more traditional method of localized virotherapy combined with systemic administration of αPD1 [3, 4]. In contrast, a recent study by Bartee et al. demonstrated that incorporation of a truncated version of the PD1 protein into the genome of oncolytic myxoma virus (MYXV) resulted in both increased efficacy and reduced toxicity in the B16/F10 melanoma model [5].

The B16/F10 model is normally immunologically naïve and therefore relatively immune to PD1-blocking monotherapies. Similar to many oncolytic viruses, treatment with MYXV broke immunological naivety and induced massive infiltration of CD8^+^ T cells. Virotherapy, however, also upregulated expression of PDL1 which severely limited anti-tumor immunotherapy. The authors demonstrated that the anti-tumor efficacy of these T cells could be released by the addition of αPD1 blocking antibodies; however, this combination therapy resulted in only incomplete efficacy. Interestingly, a recombinant MYXV which secreted a truncated form of PD1 (vPD1) displayed significantly improved efficacy compared to the combination of MYXV and αPD1 antibody treatment. This improved efficacy did not appear to be due to higher localized concentrations of truncated PD1 as only ng/ml levels of the transgene product were detected in the tumor. This suggested that using truncated PD1 to achieve PD1 blockade might represent a qualitative improvement over the use of αPD1 antibodies. The authors hypothesize that this might be due to a variety of potential mechanisms (Fig. 1), including: improved affinity of truncated PD1 to PDL1, simultaneous blockade of alternative PD1 ligands, such as PDL2, or increased diffusion of truncated PD1 through the tumor due to its smaller size. Future extrapolation of the findings from this study into other systems will likely require a conclusive demonstration of which of these mechanisms mediates the improved efficacy of truncated PD1.

**Fig. 1:**
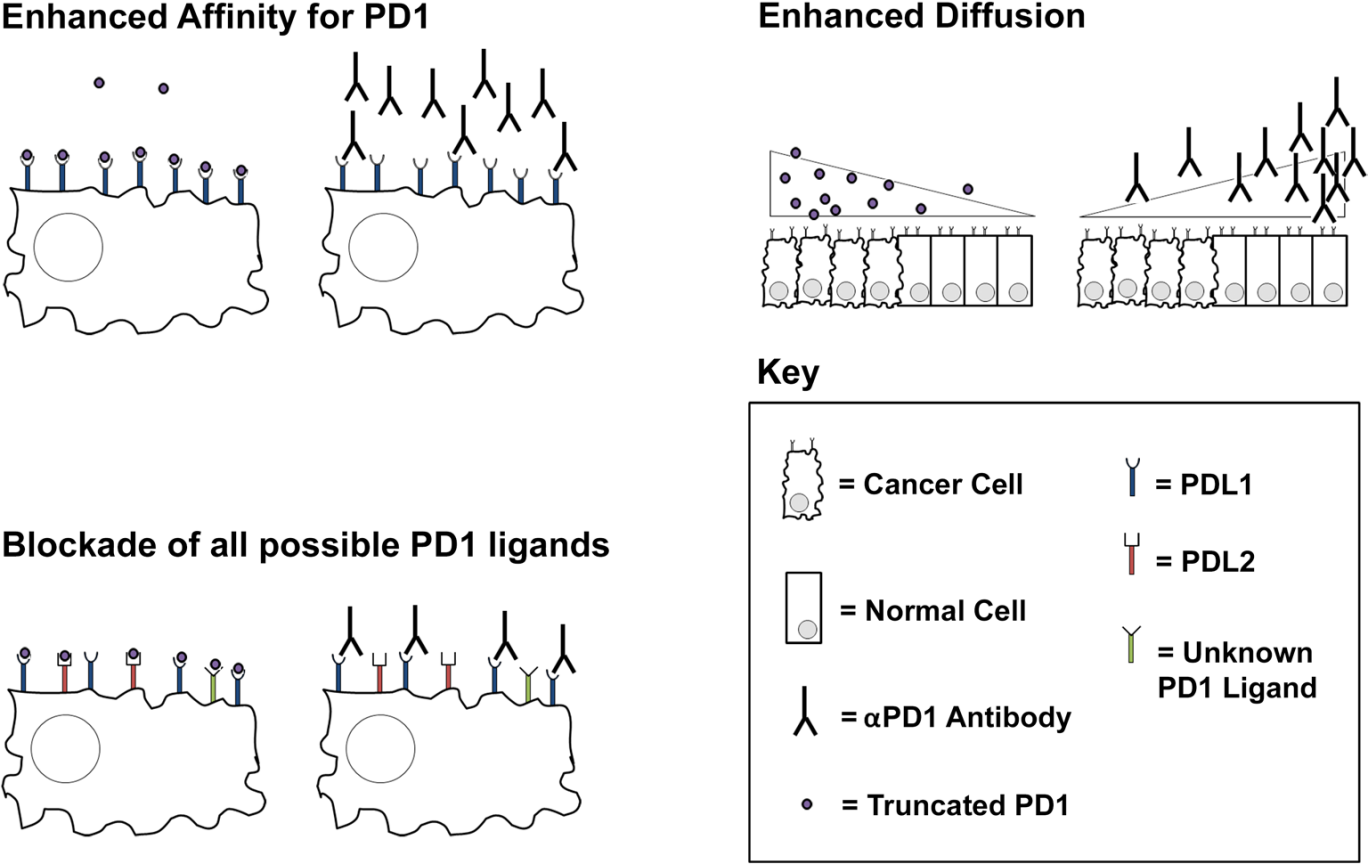
Potential mechanisms through which truncated PD1 might improve checkpoint blockade. *Enhanced affinity*: enhancements in either the binding affinity or the functional avidity of truncated PD1 compared to αPD1 antibodies could result in better saturation of PDL1 molecules on the surface of tumors cells. This would provide fewer PDL1 molecules to engage with inhibitory PD1 on the surface of activated T cell resulting in improved blockade efficacy. *Blockade of all possible PD1 ligands*: αPD1 antibodies might block interactions of PD1 with only a few possible ligands. This would allow for inhibitory signals to be sent to T cells by engagement of PD1 with unblocked ligands. In contrast, truncated PD1 should bind to all potential PD1 ligands, including those which might not be currently appreciated, thus providing a more complete blockade. *Enhanced diffusion*: αPD1 antibodies are large molecules whose diffusion into the tumor microenvironment from the vasculature is known to be inefficient. In contrast, truncated PD1 is a much smaller protein which might have improved diffusion properties. This could provide a more complete PD1 blockade by saturating a higher percentage of the tumor microenvironment with PD1-blocking reagent

Interestingly, in this same study, the authors also reported that tumor localized secretion of truncated PD1 resulted in less severe autoimmune-like toxicities compared to systemically injected αPD1 antibodies. Development of autoimmune-like toxicities in preclinical models has not often been reported following PD1 blockade [6]. The author’s observation that the combination of MYXV and systemic αPD1 antibodies induced severe, progressive alopecia in mice might provide an excellent opportunity to characterize the toxicities associated with PD1-blockade. Unfortunately, the authors were not able to completely characterize their auto-immune pathology or determine whether this reduction in auto-immune toxicity was due to tumor localization of the PD1 blockade of the use of truncated PD1. Further studies are therefore clearly needed to elucidate the mechanisms involved in using truncated PD1 to achieve PD1-blockade.

In this regards, it is interesting to note that little is known about the naturally occurring soluble splice variant of PD1 the authors based their construct on [7]. Correlative human studies have shown that serum concentrations of this variant are increased in patients suffering from a variety of inflammatory disorders including diabetes and rheumatoid arthritis suggesting it has a proinflammatory function. However, few mechanistic studies have been conducted into either the production of this splice variant or its exact role in human health. Further studies are therefore needed to fully unmask the therapeutic potential of this naturally occurring variant.

In conclusion, PD1-based checkpoint blockade is rapidly becoming a revolutionary form of cancer therapy. However, the traditional methodology of systemically injected αPD1 antibodies remains imperfect. Additional studies into alternative methods of providing PD1-blockade, such as the one by Bartee et al. [5], therefore provide an important advance into an already promising field.

## Abbreviations

PD1: programmed cell death protein 1
PDL1: programmed death ligand 1
MYXV: myxoma virus
vPD1: recombinant MYXV which secreted a truncated form of PD1
